# Topic of Influence, Methane and Microbes

**DOI:** 10.1264/jsme2.ME3204rh

**Published:** 2017-12-27

**Authors:** Hiroyuki Imachi

**Affiliations:** 1 Department of Subsurface Geobiological Analysis and Research, Japan Agency for Marine-Earth Science and Technology (JAMSTEC) 2–15 Natsushima-cho, Yokosuka, 237–0061 Japan

What is an influential study? Although there are many different aspects and opinions to consider, I will never forget the words from one of my teachers in a class a long time ago. He said that a study with a true impact produces a discovery or method that later becomes known without citation. My teacher referred to the development of the polymerase chain reaction (PCR) as an example of an influential study (37). All readers know that PCR is a method of DNA amplification that uses oligonucleotide primers and a thermostable DNA polymerase obtained from *Thermus aquaticus*, a thermophilic bacterium. Other examples of influential studies in the field of microbiology may be found in the American Society for Microbiology (ASM)’s “Significant Events in Microbiology” list, which was published to celebrate the society’s centennial. This list was first released in 1999 and included only the top 26 events; however, ASM journal editors and other members have since added more items, such that there are now approximately 300 studies listed as “Significant events in the history of microbiology” (https://www.asm.org/index.php/71-membership/archives/7852-significant-events-in-microbiologysince-1861). Readers may find some discoveries on the list that are closely related to their present research topics. I personally focused on two studies on methane: Sohngen’s 1906 discovery of methane-oxidizing bacteria (41), and Bryant and colleagues’ 1967 discovery of interspecies hydrogen transfer between hydrogen-producing organotrophic bacteria and methane-producing archaea (5). I am very familiar with these studies, but no longer cite them and have not noted them in the reference lists of any other recent studies on methane; they appear to have had such an impact that they are not cited anymore.

It has been approximately 50 and 100 years since these two great discoveries. Microbiological research subsequently revealed that most methane released into the atmosphere comes from methanogens, and also that methane-oxidizing microbes significantly contribute to control over the atmosphere release of methane (*e.g.*, 4, 14, 32). Since methane is a powerful greenhouse gas and an important energy source for human activity, many microbiological studies have been conducted on topics related to methane production and consumption. For example, advances in methane-related microbiology are among recent research on the discovery of a methanogenic archaea that generate methane directly from methoxy compounds in coal (24), the possible direct interspecies transfer of electrons by anaerobic methane-oxidizing archaea (25, 26, 38, 45), and the potential capability of methane metabolism in uncultured archaeal lineages other than conventional methanogens within the phylum *Euryarchaeota*, as predicted by metagenomic studies (8, 44). Recent issues of *Microbes and Environments* contain a number of studies on methane and microbes that are specifically relevant to methane production in, for example, rice fields, accretionary prisms, and biological wastewater treatment systems.

Rice paddy fields represent an important source of methane emissions, *i.e.*, they may contribute 10% to 20% of global methane emissions (14), and, thus, understanding the dynamics of methane in rice paddies is important. The amount of methane released into the atmosphere is tightly controlled by the balance between microbial methane production and oxidation by methane-producing archaea and methane-oxidizing bacteria, respectively. Based on the observation that increased CO_2_ levels in the atmosphere promote the production and emission of methane from rice paddies, Liu and colleagues investigated the effects of increased CO_2_ levels, elevated soil temperatures, and the absence of nitrogen fertilization on methane-producing and -oxidizing microbial populations in a free-air CO_2_ enrichment (FACE) experimental paddy field (20). Using PCR with denaturing gradient gel electrophoresis (DDGE) and quantitative PCR techniques targeting the genes *mcrA* and *pmoA*, which encode key enzymes for methane production and oxidization, they showed that the abundance of methane-producing archaea, detected as *mcrA* gene abundance, was not affected by increased CO_2_ levels and elevated soil temperatures, but by the absence of nitrogen fertilizer, leading to low abundance. In contrast, the abundance of methane-oxidizing bacteria, detected as *pmoA* gene abundance, was significantly decreased by increased CO_2_ levels and no nitrogen fertilization at the rice mid-ripening stage and also by elevated temperatures in the upper soil layer. Although several reasons for a decrease in methane-oxidizing bacteria have been considered, elevated atmospheric CO_2_ levels appear to have greatly increased the microbial biomass in soil, resulting in the depletion of O_2_ availability and a reduced abundance of methane-oxidizing bacteria. In addition to this study, other microbial ecological studies on rice paddies have been published in *Microbes and Environments* (2, 7, 12, 13, 22, 27, 28, 34, 36). In consideration of future food production and global warming, detailed research on the dynamics of methane released from rice paddies will become more important.

Accretionary prisms are mainly formed from past marine sediments scraped off a region of the oceanic plate during subduction at a convergent plate boundary. Many regions in Japan consist of accretionary prisms. Large amounts of methane are produced in the deep underground environments of accretionary prisms and then dissolve into the groundwater. Kimura and colleagues revealed that methane contained in deep aquifers of the accretionary prism obtained from coastal areas of Shizuoka Prefecture was predominantly produced by the thermal degradation of organic matter, while methane collecting in deep aquifers inland and in mountainous areas was produced by microbial activity (23). Polyphasic analyses of the chemical composition of groundwater, the carbon isotope ratio of methane, the microbial composition of 16S rRNA gene tag sequences, fluorescence *in situ* hybridization (FISH), and culture experiments led to the conclusion that microbiologically produced methane is derived from the symbiotic relationship between hydrogenogenic fermenting bacteria and hydrogenotrophic methanogenic archaea. Based on these findings and their previous result (18), Kimura and colleagues reported that accretionary prisms are an energy-producing system that are useful for establishing natural factories for methane production, hydrogen production, and electricity generation (3). This research has provided a scientific basis for the future utilization of the natural methane production ecosystem in accretionary prisms by local governments and companies.

The symbiotic relationship between fermenting bacteria and methane-producing archaea is generally observed in diverse anaerobic environments, such as in the deep aquifers of the accretionary prisms described above. Anaerobic digestion (AD) is a representative technology that makes use of the symbiotic relationships between anaerobic microorganisms (21, 30, 39, 43). AD technology is widely used in the treatment of wastewater and has already been extensively examined (1, 11, 29, 46). In *Microbes and Environments*, a study was published by Narihiro and colleagues on comparative genomics of three strains belonging to the family *Syntrophomonadaceae*, the members of which are frequently observed in AD systems. These strains are fermenting bacteria (so-called syntrophs) that grow symbiotically with methanogens via interspecies hydrogen (and/or formate) transfer (31). Based on a comparative genomic analysis of three syntrophs from the *Syntrophomonadaceae* family that break down branched-chain fatty acids (BCFAs) such as 2-methylbutyrate and isobutyrate, the *Syntrophomonadaceae* strains were found to have unique β-oxidation systems of BCFAs and short-chain fatty acids for syntrophic substrate oxidation as well as unique energy-conserving electron transport systems.

Most of the microbiological investigations conducted on AD technology have focused on members of *Bacteria* and *Archaea* due to their large populations in AD systems. However, Hirakara and co-workers reported eukaryotic populations (protists) (9, 10). Protists are known to be predators of prokaryotes and have a significant impact on the composition and function of the co-existing prokaryotic population. However, the effects of protists on prokaryotic communities in anaerobic environments have not yet been examined in detail. Hirakata and colleagues prepared two upflow anaerobic sludge blanket (UASB) reactors and performed co-cultivations of the anaerobic ciliates *Metopus* and *Caenomorpha* with granular sludge in one of the reactors. The USAB reactor co-cultivated with anaerobic ciliates exhibited stronger methane production activity than the UASB reactor without anaerobic ciliates. Based on these findings, the authors stated that the effects of predation by protists in AD systems have mostly been overlooked, and that the influence of predation by protists needs to be considered in order to obtain a better understanding of the structure and function of a prokaryotic community.

As discussed herein, *Microbes and Environments* has published a number of studies relevant to methane (*e.g.*, 6, 15–17, 19, 33, 35, 40, 42). In the near future, I hope that some of these studies published in *Microbes and Environments* will contribute to clarifying important social issues associated with methane and microbes, such as global warming and energy supply, and will become “influential studies” with no need for citation.
